# Mitomycin C potentiates metronidazole activity in resistant *Trichomonas vaginalis* through suppression of thioredoxin reductase

**DOI:** 10.1016/j.ijpddr.2026.100661

**Published:** 2026-07-18

**Authors:** Yuan-Ming Yeh, Po-Jung Huang, Yu-Lo Wu, Chun-Hsien Chen, Pei-Yun Chen, Yu-Tzu Hsu, Wei-Hung Cheng

**Affiliations:** a Genomic Medicine Core Laboratory, Chang Gung Memorial Hospital, Linkou, Taiwan; b Graduate Institute of Health Industry Technology, Chang Gung University of Science and Technology, Taoyuan, Taiwan; c Graduate Institute of Biomedical Sciences, College of Medicine, Chang Gung University, Taoyuan, Taiwan; d Department of Biomedical Sciences, College of Medicine, Chang Gung University, Guishan Dist., Taoyuan City, Taiwan; e Department of Parasitology, College of Medicine, National Cheng Kung University, Tainan, Taiwan; f Department of Parasitology, College of Medicine, Chang Gung University, Guishan Dist., Taoyuan, Taiwan; g Institute of Basic Medical Sciences, College of Medicine, National Cheng Kung University, Tainan, Taiwan; h Department of Medicine, College of Medicine, National Cheng Kung University, Tainan, Taiwan

**Keywords:** *Trichomonas vaginalis*, Metronidazole resistance, Ferroptosis, Thioredoxin reductase, Mitomycin C, Synergic treatment

## Abstract

Metronidazole (MTZ) remains the first-line therapy for trichomoniasis; however, increasing resistance in *Trichomonas vaginalis* threatens treatment efficacy. Because iron availability enhances MTZ sensitivity, we investigated whether MTZ-induced parasite death involves ferroptosis-related mechanisms and explored alternative pathways contributing to drug resistance. Exposure of metronidazole-sensitive and -resistant isolates to ferroptosis inducers reduced parasite viability, yet neither ferroptosis inhibition nor iron chelation restored survival, and lipid peroxidation was not detected. These findings indicate that MTZ-associated cytotoxicity does not proceed via canonical ferroptotic pathways in *T. vaginalis*. Instead, resistant parasites exhibited elevated expression of thioredoxin-associated transcripts, consistent with enhanced redox defense capacity. Functional suppression of thioredoxin reductase (TrxR) activity by mitomycin C (MMC) significantly enhanced MTZ-mediated killing and produced a synergistic antiparasitic effect in resistant isolates. Although selective TrxR inhibition alone was insufficient to fully reproduce this phenotype, these findings implicate thioredoxin-dependent redox regulation as a contributor to MTZ susceptibility. Combination treatment was accompanied by selective metabolic reprogramming, including upregulation of pentose phosphate pathway genes linked to NADPH generation. Collectively, our results demonstrate that MTZ induces a non-ferroptotic form of cell death and identify thioredoxin-dependent redox buffering as a resistance-associated vulnerability. Targeting redox homeostasis through rational combination therapy may represent a mechanistically guided strategy to overcome metronidazole resistance in trichomoniasis.

## Highlights

• Canonical ferroptosis is not induced in *Trichomonas vaginalis*.
• Thioredoxin system is upregulated in metronidazole-resistant strains.
• Mitomycin C synergizes with metronidazole against resistant parasites.

## Graphical abstract

**Figure:**
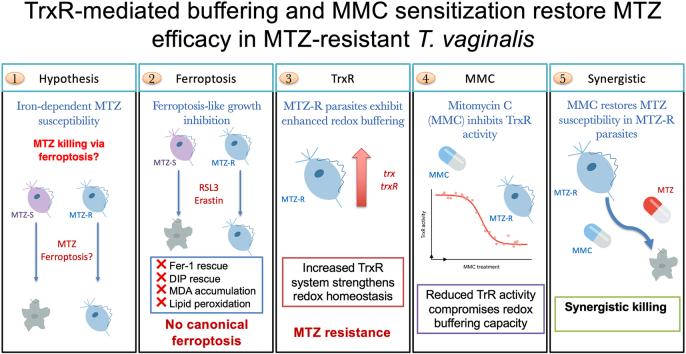

## 1 Introduction

*Trichomonas vaginalis* is one of the most prevalent non-viral sexually transmitted protozoan pathogens worldwide (Mercer and Johnson, 2018; Garber, 1998). Its infection rate in certain regions, including parts of South America and Africa, exceeds that of gonorrhea and syphilis. The parasite primarily colonizes the human urogenital tract, infecting both males and females. Notably, approximately 50% of infected women and 75% of infected men remain asymptomatic, serving as potential reservoirs for transmission. In women, infection is often associated with vaginitis, increased vaginal discharge, infertility, pelvic inflammatory disease, and an elevated risk of HIV acquisition and cervical cancer (Mercer and Johnson, 2018).

The genome of *T. vaginalis* is unusually large (∼160 Mb), with a highly repetitive structure and extensive gene duplication, contributing to remarkable genomic plasticity and adaptability (Carlton et al., 2007). The parasite lacks conventional mitochondria and instead possesses hydrogenosomes, specialized organelles involved in energy metabolism and activation of 5-nitroimidazole drugs such as metronidazole (MTZ) (Tachezy et al., 2022). Hydrogenosomes contain enzymes such as pyruvate:ferredoxin oxidoreductase (PFOR) and iron–sulfur proteins, which are critical for anaerobic metabolism and drug activation (Leitsch et al., 2009). In addition, *T. vaginalis* exhibits an atypical lipid composition, relying largely on host-derived lipids due to its limited capacity for *de novo* lipid synthesis. The presence of polyunsaturated fatty acids and diverse phospholipid species in the parasite membrane suggests potential susceptibility to oxidative lipid damage.

MTZ, a 5-nitroimidazole compound, has served as the cornerstone of trichomoniasis treatment since its development in 1959 and is currently listed as an essential medicine by the World Health Organization (Leitsch, 2019). As a prodrug, MTZ is activated under low-oxygen conditions through reduction by parasite redox enzymes, generating reactive intermediates and reactive oxygen species (ROS) that damage DNA and proteins (Leitsch et al., 2009). Resistance to MTZ, however, has become an increasing clinical concern, with reported prevalence ranging from 2.5% to 9.6% in clinical isolates (Cudmore et al., 2004; Kirkcaldy et al., 2012; Upcroft and Upcroft, 2001). Resistant strains often display cross-resistance to other 5-nitroimidazole derivatives, highlighting the urgent need for alternative therapeutic strategies.

Iron plays a dual role in *T. vaginalis* biology. It is essential for parasite growth, energy metabolism, and virulence, influencing enzymes such as PFOR and hydrogenase and regulating the expression of adhesion proteins (Sutak et al., 2008). At the same time, intracellular iron accumulation has been shown to correlate with increased MTZ susceptibility, and supplementation with iron can reduce the IC_50_ of MTZ, suggesting a functional interaction between iron homeostasis and drug sensitivity (Argaez-Correa et al., 2019; Elwakil et al., 2017; Sutak et al., 2008). Given that iron is a central mediator of ferroptosis—a regulated form of cell death characterized by iron-dependent lipid peroxidation—this observation raises the possibility that MTZ-induced cytotoxicity may involve ferroptosis-like mechanisms (Dixon et al., 2012; Conrad and Pratt, 2019; Tang et al., 2021).

Ferroptosis is defined by the accumulation of lipid peroxides in polyunsaturated phospholipids, driven by iron-catalyzed reactions and failure of antioxidant systems such as glutathione peroxidase 4 (GPX4) (Dixon et al., 2012; Conrad and Pratt, 2019; Jiang et al., 2021; Stockwell, 2022; Tang et al., 2021). However, *T. vaginalis* lacks a canonical glutathione-based antioxidant system and instead relies primarily on alternative redox pathways, including the thioredoxin and peroxiredoxin systems (Ellis et al., 1994; Müller et al., 2003; Lu and Holmgren, 2014). The thioredoxin–peroxiredoxin cascade has been proposed as a core antioxidant network in this parasite and may represent a potential therapeutic vulnerability (Müller et al., 2003).

Mitomycin C (MMC), a clinically used agent known to induce redox imbalance and DNA damage, has also been reported to interfere with thioredoxin reductase (TrxR) activity and thioredoxin-mediated redox control (Tomasz, 1995; Paz et al., 2012). These properties make MMC a suitable pharmacological probe to examine the functional relevance of the thioredoxin system in drug-resistant *T. vaginalis*.

Previous transcriptomic analyses revealed that MTZ-resistant strains exhibit upregulation of antioxidant-related pathways compared with sensitive strains following MTZ exposure (Huang et al., 2021). These findings further support the notion that enhanced redox defense mechanisms may contribute to drug resistance. Moreover, lipid analyses have shown that *T. vaginalis* membranes contain unusual acyl lipids and unsaturated phospholipids, providing potential substrates for oxidative deterioration (Guschina et al., 2009). This enrichment of unsaturated phospholipids supports the hypothesis that lipid peroxidation and redox imbalance may contribute to drug-induced parasite death (Yin et al., 2011).

Based on these observations, this study aimed to determine whether MTZ-induced killing of *T. vaginalis* involves ferroptosis-related mechanisms and to identify alternative redox pathways contributing to drug resistance. Furthermore, we explored the potential of targeting the thioredoxin system using mitomycin C as a combinatorial strategy to enhance MTZ efficacy against resistant strains.

## 2 Materials and methods

### 2.1 Parasite strains and culture conditions

Metronidazole-sensitive (*T. vaginalis* ATCC 30236) and metronidazole-resistant (*T. vaginalis* ATCC 50143) strains were obtained from the American Type Culture Collection (ATCC), where their metronidazole susceptibility phenotypes are documented. These designations are consistent with previously reported susceptibility profiles, including our prior study (Huang et al., 2021). Parasites were maintained in YI-S (Yeast Iron–Horse serum) medium supplemented with 10% horse serum and ferric ammonium citrate at 37 °C and subcultured at mid-log phase. All experiments were conducted under identical routine culture conditions without deliberate modification of oxygen availability. Both strains exhibited comparable growth rates and maximal cell densities under the culture conditions used in this study.

### 2.2 Drug preparation and treatment conditions

Ras-selective lethal 3 (RSL3), erastin, and ferrostatin-1 (Fer-1) were purchased from MedChemExpress. Mitomycin C (MMC), metronidazole (MTZ), and 2,2-dipyridyl (DIP) were obtained from Sigma-Aldrich. Ferroptosis-related compounds were dissolved in DMSO to prepare 10 mM stock solutions and stored at −20 °C. MTZ was dissolved in sterile distilled water. C11-BODIPY 581/591 (Sigma-Aldrich) was prepared in DMSO as a 5 mM stock solution. RSL3 and erastin concentrations were selected based on previously published ferroptosis studies (Bogacz et al., 2018; Das et al., 2020; Silvestre et al., 2023), whereas the Fer-1 and DIP concentrations were selected based on previous studies of ferroptosis and iron regulation in *T. vaginalis* (Kose et al., 2022; Cheng et al., 2015).

For all drug treatment experiments, parasites were harvested at mid-log phase and seeded at a density of 1 × 10^6^ cells/mL in 10 mL YI-S medium in sterile culture tubes prior to compound exposure. Parasites were then treated with the indicated concentrations of compounds for 12–18 h depending on the assay. Vehicle controls containing equivalent solvent concentrations were included in all experiments, and the final DMSO concentration (0.2%) was kept constant across treatments.

### 2.3 Viability assessment by trypan blue exclusion

Parasite viability following drug treatment was determined using Trypan blue exclusion. Treated cultures were gently mixed, diluted in PBS, and stained with 0.4% Trypan blue for 1–2 min. Samples were loaded onto a hemocytometer and observed under a light microscope at 10× magnification. Live parasites appeared unstained and motile, whereas nonviable parasites were stained dark blue. Viability was calculated as the percentage of live parasites over total counted parasites. All experiments were performed in three independent biological replicates. The 18-h incubation period was selected based on preliminary optimization experiments and previous studies of *T. vaginalis* growth kinetics, as this time point allowed reliable detection of drug-induced effects while minimizing secondary influences associated with prolonged culture.

### 2.4 Lipid peroxidation assays

Lipid peroxidation was evaluated using both a colorimetric malondialdehyde (MDA) assay (Abcam) and the fluorescent probe C11-BODIPY 581/591 (Invitrogen) (Yin et al., 2011).

For MDA measurement, parasites were lysed in PBS containing 0.5% Triton X-100 after 16 h of drug exposure. Samples were reacted with MDA color reagent according to the manufacturer's instructions and absorbance was measured at 532 nm using a microplate reader.

For fluorescence-based detection, parasites were incubated with 10 μM C11-BODIPY for 30 min at 37 °C following drug treatment. Samples were analyzed using flow cytometry (FACS Canto II or CytoFlex), and fluorescence shifts from red to green emission were quantified.

HeLa and Caco-2 cells were used as positive controls to validate ferroptosis and lipid peroxidation detection methods (Supplementary Fig. 1).

### 2.5 RNA extraction and quantitative RT-PCR

Total RNA was extracted 18 h after drug treatment using TRI Reagent (Invitrogen) and purified with a commercial RNA purification kit (Direct-zol RNA miniprep, Zymo Research). RNA quality was assessed by A260/280 ratio. cDNA synthesis was performed using reverse transcriptase master mix (MorreRT Master mix, MORREBIO).

Quantitative PCR was performed using SYBR Green Master Mix (Promega) on a StepOne System (Applied Biosystems). Gene expression levels were normalized to the reference gene ribosomal protein gene L8 (TVAGG3_0062590) and calculated using the 2^−ΔΔCt method. Primer sequences are listed in Supplementary Table 1. Each experiment included three biological replicates and technical triplicates.

Target genes included thioredoxin family members, peroxiredoxins, glycolysis-related genes, pentose phosphate pathway genes, and MTZ metabolism-related genes.

### 2.6 Drug combination and synergy analysis

To evaluate the combinational effects of MTZ and MMC, parasites were exposed to five concentrations of each drug (0, 5, 10, 20, 40 μM) in a 5 × 5 matrix. Each condition was tested in triplicate in three independent experiments. Parasite viability was determined by Trypan blue exclusion and expressed as percentage survival relative to the vehicle control.

The normalized survival data were used as input for SynergyFinder 3.0 analysis using the Loewe additivity model (Ianevski et al., 2022). Synergy scores >10 were considered indicative of synergistic interaction.

### 2.7 Thioredoxin reductase activity assay

TrxR activity was measured using a DTNB-based colorimetric assay kit (Abcam, ab83463). Parasites (2–5 × 10^6^ cells) were harvested 18 h after drug treatment, lysed in TrxR assay buffer on ice, and centrifuged at 10,000 × g for 20 min at 4 °C. Supernatants were collected for analysis.

Fifty microliters of lysate were added to a 96-well plate. Background activity was determined using the TrxR inhibitor provided in the kit, while parallel wells without inhibitor represented total activity. The reaction was initiated by adding assay buffer containing DTNB and NADPH to a final volume of 100 μL.

Absorbance at 412 nm was recorded kinetically for 60 min using a microplate reader. A TNB standard curve (0–50 nmol/well) was used to convert absorbance to product formation, and TrxR activity was calculated based on reaction rate according to the manufacturer's instructions.

### 2.8 Statistical analysis

All data are presented as mean ± standard deviation from three independent experiments. Statistical analysis was performed using one-way ANOVA followed by Tukey's multiple comparison test. ∗p < 0.05; ∗∗p < 0.01; ∗∗∗p < 0.001; ∗∗∗∗p < 0.0001; ns, not significant.

## 3 Results

### 3.1 Ferroptosis inducers reveal differential stress tolerance between MTZ-S and MTZ-R parasites

Intracellular iron availability influences MTZ susceptibility in *T. vaginalis*, raising the possibility that MTZ-induced parasite death may involve ferroptosis-related processes. Because ferroptosis is an iron-dependent form of regulated cell death, we investigated whether ferroptosis-like responses could be induced in this protozoan. MTZ-sensitive (MTZ-S) and -resistant (MTZ-R) parasites were exposed to the ferroptosis inducers RSL3 or erastin, and parasite growth was monitored over time.

**Fig. 1:**
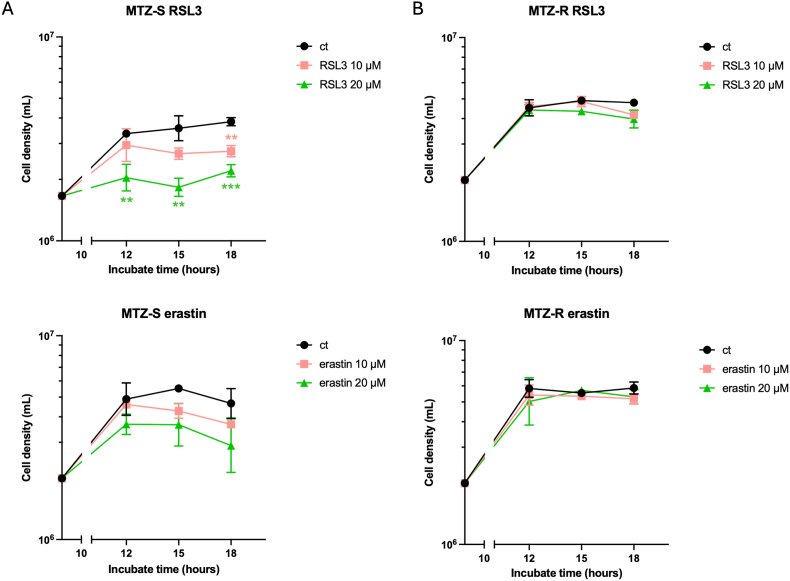
MTZ-resistant parasites exhibit reduced susceptibility to ferroptosis-inducing agents. (A) Growth curves of metronidazole-sensitive (MTZ-S) parasites treated with RSL3 and erastin (10 or 20 μM). (B) Growth curves of metronidazole-resistant (MTZ-R) parasites treated with RSL3 and erastin (10 or 20 μM). ct, control groups received equivalent volumes of DMSO. Parasites at mid-log phase (1 × 10^6^ cells/mL) were incubated for the indicated time points. Cell density was determined by hemocytometer counting. Data represent mean ± SD from three independent experiments. Statistical significance was evaluated using a Student's t-test.

**Fig. 2:**
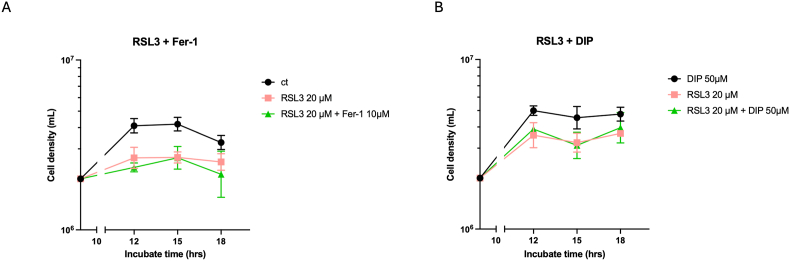
Ferroptosis inhibition and iron chelation do not restore parasite viability. Parasite viability following treatment with RSL3 (20 μM) in the presence or absence of ferrostatin-1 (Fer-1, 10 μM) (A) or 2,2-dipyridyl (DIP, 50 μM) (B). Cell density was determined by the trypan blue exclusion assay. ct, control groups received equivalent volumes of the corresponding vehicle (DMSO for RSL3 and Fer-1; sterile water for DIP).

Both agents reduced the viability of MTZ-S parasites in a dose-dependent manner. In contrast, MTZ-R parasites showed minimal growth inhibition under identical treatment conditions (Fig. 1A and B). To determine whether the observed cytotoxicity followed canonical ferroptosis, parasites were co-treated with the ferroptosis inhibitor ferrostatin-1 or the iron chelator 2,2-dipyridyl (DIP). Neither intervention restored parasite survival following exposure to ferroptosis inducers (Fig. 2).

**Fig. 3:**
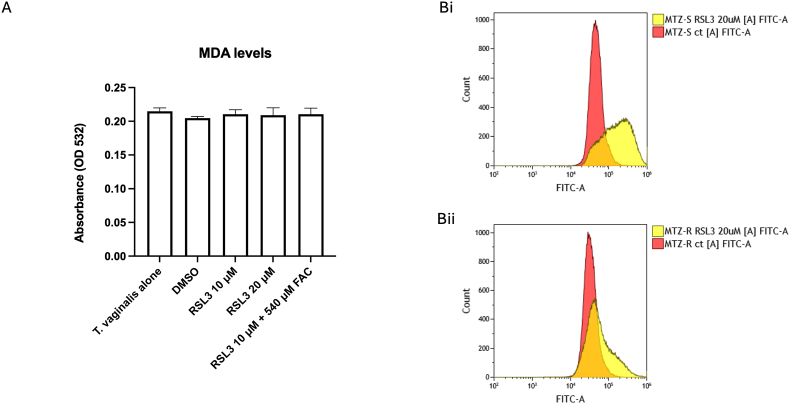
RSL3 treatment does not induce lipid peroxidation in *T. vaginalis.* (A) Lipid peroxidation was quantified by measuring malondialdehyde (MDA) levels using a thiobarbituric acid reactive substances (TBARS) assay. The MTZ-S parasites were treated with RSL3 (10 or 20 μM) in the presence or absence of ferric ammonium citrate (FAC, 540 μM) as indicated. Absorbance was measured at 532 nm and expressed as OD values. Data represent mean ± SD from three independent experiments. (B) Lipid ROS levels were assessed by flow cytometry using the oxidation-sensitive fluorescent probe C11-BODIPY 581/591. Representative histograms of MTZ-S (Bi) and MTZ-R (Bii) parasites treated with RSL3 (20 μM) are shown. Fluorescence intensity was detected in the FITC channel (FITC-A). Red curves represent untreated controls; yellow curves represent RSL3-treated samples.

Analysis of lipid peroxidation showed no significant increase in MDA levels (Fig. 3A). Consistent with this finding, assessment of lipid ROS by C11-BODIPY staining revealed only a marginal fluorescence shift following RSL3 treatment in both MTZ-S (Fig. 3Bi) and MTZ-R (Fig. 3Bii) parasites, indicating that any lipid peroxidation induced under these conditions was minimal and remained below the threshold of robust biochemical detection.

Collectively, these results demonstrate that although ferroptosis inducers are cytotoxic to *T. vaginalis*, the resulting cell death does not follow classical ferroptotic mechanisms (Dixon et al., 2012; Conrad and Pratt, 2019; Stockwell, 2022). Notably, under the tested conditions, ferroptosis-inducing compounds produced more pronounced growth inhibition in MTZ-S parasites than in MTZ-R parasites, suggesting differential stress tolerance between the two isolates. Because both MTZ and ferroptosis inducers can disrupt cellular redox homeostasis, these observations suggest that these compounds may elicit partially overlapping cellular stress responses in *T. vaginalis*. Such differential susceptibility prompted us to further examine whether antioxidant pathways contribute to parasite responses under drug-induced stress.

### 3.2 Thioredoxin-associated transcripts are induced by drug-associated oxidative stress in *T. vaginalis*

In light of the differential susceptibility of MTZ-S and MTZ-R parasites to ferroptosis-inducing compounds, we next examined whether antioxidant pathways are activated in *T. vaginalis* under drug-induced stress. Because this parasite lacks a conventional glutathione-dependent antioxidant system, it relies primarily on the thioredoxin (Trx)–peroxiredoxin redox network to maintain intracellular redox homeostasis (Ellis et al., 1994; Müller et al., 2003). We therefore analyzed the transcriptional responses of Trx-associated genes following drug exposure.

**Fig. 4:**
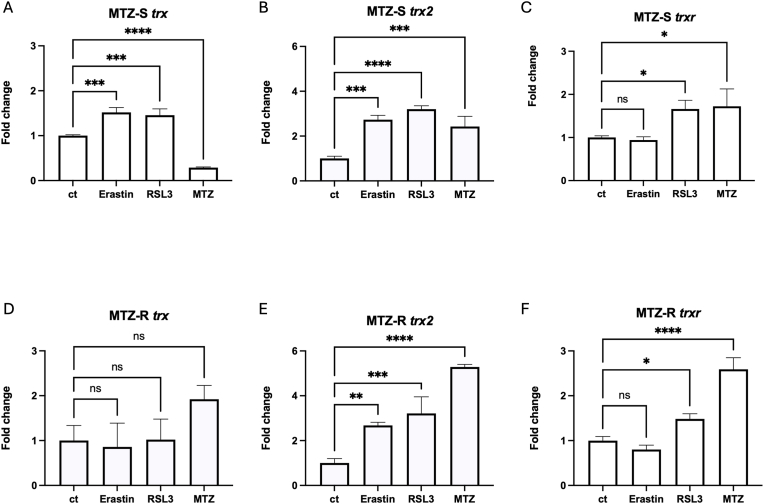
Thioredoxin-associated genes show differential stress responses in MTZ-sensitive and resistant parasites. Expression levels of thioredoxin (*trx*) (TVAGG3_0939120), *trx2* (TVAGG3_0657800), and thioredoxin reductase (*trxr*) (TVAGG3_0344710) were measured in MTZ-S (A–C) and MTZ-R (D–F) *T. vaginalis* cells following treatment with erastin (20 μM), RSL3 (20 μM), or MTZ (20 μM) for 18 h. Relative transcript levels were determined using the 2^−^ΔΔCt method and normalized to untreated controls using ribosomal protein L8 (TVAGG3_0062590) as the reference gene. Data represent the mean ± SD of three independent experiments. ct, vehicle controls received the same volume of DMSO as drug-treated groups. Statistical significance was determined by one-way ANOVA followed by multiple-comparison testing. ∗p < 0.05; ∗∗p < 0.01; ∗∗∗p < 0.001; ∗∗∗∗p < 0.0001; ns, not significant.

In MTZ-S parasites, treatment with the ferroptosis inducers erastin or RSL3 resulted in consistent induction of Trx and Trx2 transcripts. In contrast, significant upregulation of TrxR was observed only in RSL3-and MTZ-treated parasites, whereas erastin treatment did not significantly alter TrxR expression (Fig. 4A–C). These responses indicate activation of thioredoxin-mediated antioxidant defenses under drug-induced stress conditions.

A similar pattern of Trx-system activation was observed in the MTZ-R isolate. However, the magnitude of transcriptional responses differed for specific components of the pathway. While Trx expression showed relatively limited responsiveness, TrxR transcripts were strongly induced following MTZ exposure (Fig. 4D–F). This enhanced induction of TrxR in the resistant isolate suggests that MTZ-R parasites may rely more heavily on TrxR-mediated redox buffering during drug challenge.

Collectively, these results indicate that exposure to MTZ and ferroptosis-inducing agents triggers oxidative stress responses in *T. vaginalis*, leading to activation of thioredoxin-associated transcripts. The more pronounced induction of TrxR observed in the MTZ-R isolate suggests that the thioredoxin system is engaged during MTZ challenge and may contribute to parasite adaptation to drug-induced redox stress.

### 3.3 Functional inhibition of TrxR underlies MTZ–MMC synergistic killing

To determine whether the transcriptional induction of TrxR correlates with changes in enzyme activity, we next measured TrxR activity in MTZ-R parasites following drug treatment. MMC was used as a pharmacological probe because it has been reported to interfere with TrxR activity and induce DNA damage (Tomasz, 1995; Paz et al., 2012). TrxR activity was measured in the MTZ-R isolate using a DTNB-based assay.

**Fig. 5:**
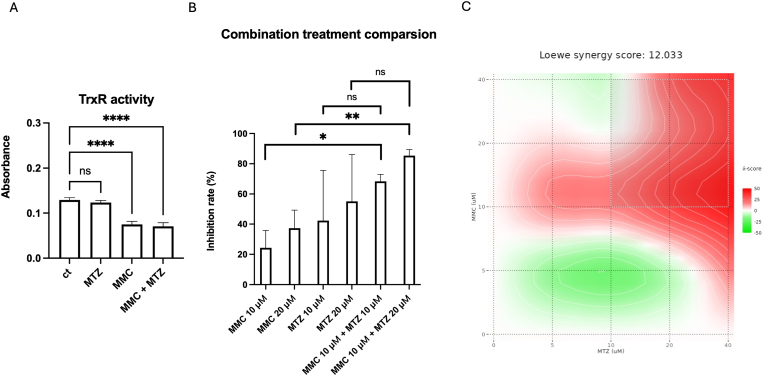
Mitomycin C suppresses TrxR activity and synergizes with MTZ in resistant parasites. (A) Thioredoxin reductase (TrxR) activity measured using a DTNB-based colorimetric assay following treatment with MTZ (20 μM), MMC (10 μM), or combination for 18 h. Absorbance at 412 nm was monitored kinetically as an indicator of TrxR activity. Data represent mean ± SD from three independent experiments. (B) Growth inhibition of MTZ-R parasites following treatment with MMC (10 or 20 μM), MTZ (10 or 20 μM), or the indicated combination treatments for 18 h. Inhibition rates were calculated relative to untreated controls. Data represent mean ± SD from three independent experiments. Statistical significance was determined by one-way ANOVA followed by multiple-comparison testing. ∗p < 0.05; ∗∗p < 0.01; ns, not significant. (C) Drug combination matrix of MTZ and MMC in MTZ-R parasites. Parasites were treated with a 5 × 5 concentration matrix (0–40 μM each drug) for 18 h ct, vehicle-treated controls receiving equivalent volumes of DMSO (for MMC) and sterile water (for MTZ). Growth inhibition was calculated relative to vehicle-treated controls and analyzed using SynergyFinder 3.0 using the Loewe additivity model. The heatmap displays Loewe synergy scores, where positive values indicate synergistic interactions and negative values indicate antagonistic interactions.

MTZ treatment alone did not significantly affect TrxR activity. In contrast, MMC significantly reduced TrxR enzymatic activity, and a similar reduction was observed under MMC–MTZ co-treatment conditions (Fig. 5A).

To determine whether this functional inhibition translates into enhanced antiparasitic efficacy, we performed combination viability assays. The results showed that MTZ-resistant parasites were less sensitive to low concentrations of MMC than MTZ-sensitive parasites (Fig. S2), indicating that the enhanced activity observed during combination treatment cannot be explained solely by the direct cytotoxicity of MMC. Moreover, combination treatment significantly enhanced inhibition compared with MMC monotherapy, while inhibitory effects also showed an increasing trend relative to MTZ monotherapy (Fig. 5B). SynergyFinder analysis using the Loewe additivity model yielded a synergy score greater than 10 (Fig. 5C), indicating a synergistic interaction (Ianevski et al., 2022; Ma and Motsinger-Reif, 2019).

These findings indicate that suppression of TrxR activity is associated with increased MTZ sensitivity in the resistant parasite. However, treatment with the selective TrxR inhibitor auranofin (AF) did not significantly inhibit MTZ-R parasite growth either alone or in combination with MTZ (Fig. S3), suggesting that selective TrxR inhibition alone is insufficient to fully reproduce the MMC–MTZ phenotype.

### 3.4 Combination treatment induces selective metabolic reprogramming associated with redox and MTZ activation pathways

The finding that AF failed to reproduce the MMC–MTZ phenotype in the MTZ-R isolate suggested that selective TrxR inhibition alone is insufficient to explain the observed synergistic interaction. We therefore investigated whether MMC–MTZ co-treatment induced broader metabolic adaptations by examining genes involved in glycolysis, the pentose phosphate pathway (PPP), and MTZ-associated redox metabolism. These pathways were selected because they contribute to cellular redox homeostasis, NADPH generation, and electron-transfer reactions associated with TrxR function and MTZ activation.

**Fig. 6:**
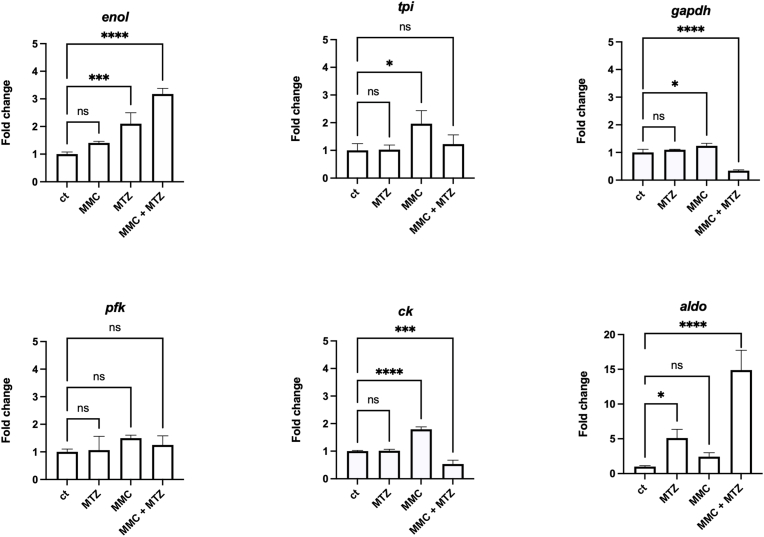
MTZ–MMC co-treatment selectively alters glycolytic gene expression. Expression levels of enolase (*enol*) (TVAGG3_1048680), triose phosphate isomerase (*tpi*) (TVAGG3_0815950), fructose-bisphosphate aldolase (*aldo*) (TVAGG3_0120590), glyceraldehyde-3-phosphate dehydrogenase (gapdh) (TVAGG3_0303510), phosphofructokinase (*pfk*) (TVAGG3_0322530), and creatine kinase (*ck*) (TVAGG3_0581060) were measured in MTZ-R *T. vaginalis* following treatment with MTZ (40 μM), MMC (10 μM), or co-treatment for 18 h. Relative transcript levels were determined using the 2^−^ΔΔCt method and normalized to untreated controls using ribosomal protein L8 (TVAGG3_0062590) as the reference gene. ct, control groups received equivalent volumes of the corresponding vehicle (DMSO for MMC; sterile water for MTZ). Data represent the mean ± SD of three independent experiments. Statistical significance was determined by one-way ANOVA. ∗p < 0.05; ∗∗p < 0.01; ∗∗∗p < 0.001; ∗∗∗∗p < 0.0001; ns, not significant.

Among glycolytic genes, enolase (*enol*) and fructose-bisphosphate aldolase (*aldo*) were significantly upregulated under combination treatment, whereas triose phosphate isomerase (*tpi*) and phosphofructokinase (*pfk*) exhibited modest or non-significant changes (Fig. 6). Notably, glyceraldehyde-3-phosphate dehydrogenase (*gapdh*) and creatine kinase (*ck*) were downregulated following co-treatment. These findings indicate that glycolytic modulation under MTZ–MMC exposure is selective rather than uniformly increased, suggesting metabolic rebalancing rather than generalized glycolytic activation.

**Fig. 7:**
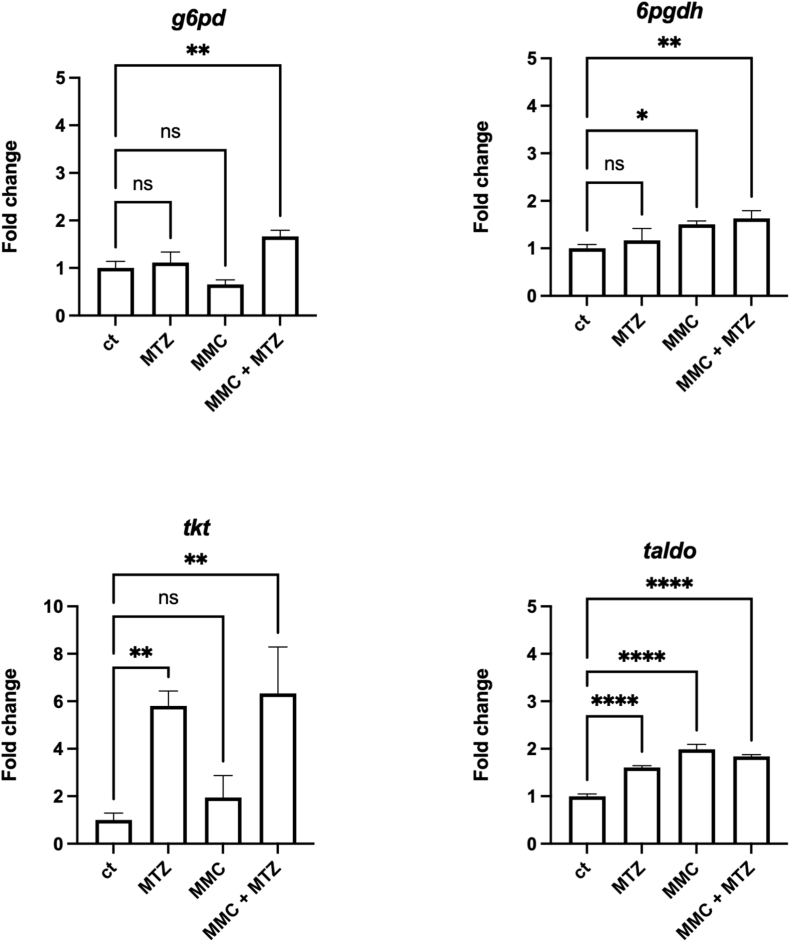
MTZ–MMC treatment induces pentose phosphate pathway genes linked to NADPH generation. Expression levels of glucose-6-phosphate dehydrogenase (*g6pd*) (TVAGG3_0205560), 6-phosphogluconate dehydrogenase (*6pgdh*) (TVAGG3_0982660), transketolase (*tkt*) (TVAGG3_0961100), and transaldolase (*taldo*) (TVAGG3_0155570) were measured in MTZ-R *T. vaginalis* parasites following treatment with MTZ (40 μM), MMC (10 μM), or co-treatment for 18 h. Relative transcript levels were determined using the 2^−^ΔΔCt method and normalized to untreated controls using ribosomal protein L8 (TVAGG3_0062590) as the reference gene. ct, control groups received equivalent volumes of the corresponding vehicle (DMSO for MMC; sterile water for MTZ). Data represent the mean ± SD of three independent experiments. Statistical significance was determined by one-way ANOVA. ∗p < 0.05; ∗∗p < 0.01; ∗∗∗p < 0.001; ∗∗∗∗p < 0.0001; ns, not significant.

In contrast, genes involved in the PPP displayed a more consistent pattern. Transketolase (*tkt*) and transaldolase (*taldo*), which mediate carbon rearrangement reactions within the non-oxidative PPP, were markedly upregulated under combination treatment, accompanied by moderate increases in glucose-6-phosphate dehydrogenase (*g6pd*) and 6-phosphogluconate dehydrogenase (*6pgdh*), two key enzymes responsible for NADPH generation in the oxidative PPP (Fig. 7). Because the PPP is a major source of NADPH required for thioredoxin-mediated redox control and nucleotide biosynthesis, these changes are consistent with increased demand for reducing equivalents and DNA repair precursors under combined redox and genotoxic stress (Patra and Hay, 2014; Stincone et al., 2015; Stockwell, 2022).

**Fig. 8:**
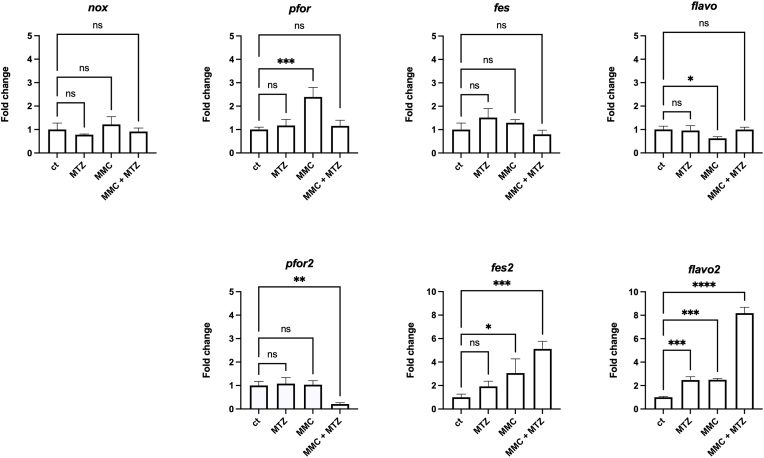
Combination treatment modulates MTZ-associated metabolic and redox genes. Expression levels of NADH oxidase (*nox*) (TVAGG3_0548570), pyruvate:ferredoxin oxidoreductase (*pfor*) (TVAGG3_0998520), *pfor2* (TVAGG3_0751360), 4Fe-4S binding domain containing protein (*fes*) (TVAGG3_0170920), *fes2* (TVAGG3_0517720), and flavodoxin (*flavo*) (TVAGG3_0565200), and *flavo2* (TVAGG3_0389530) were measured in MTZ-R *T. vaginalis* parasites following treatment with MTZ (40 μM), MMC (10 μM), or combination treatment for 18 h. Relative transcript levels were determined using the 2^−^ΔΔCt method and normalized to untreated controls using ribosomal protein L8 (TVAGG3_0062590) as the reference gene. ct, control groups received equivalent volumes of the corresponding vehicle (DMSO for MMC; sterile water for MTZ). Data represent the mean ± SD of three independent experiments. Statistical significance was determined by one-way ANOVA. ∗p < 0.05; ∗∗p < 0.01; ∗∗∗p < 0.001; ∗∗∗∗p < 0.0001; ns, not significant.

We further analyzed genes associated with MTZ activation pathways, including *pfor*, Fe–S cluster components, and flavin-associated enzymes (Fig. 8). Combination treatment resulted in significant upregulation of 4Fe-4S binding domain containing protein (*fes2*) and flavodoxin 2 (*flavo2*), alongside downregulation of *pfor2*. These alterations suggest selective modulation of hydrogenosomal redox enzymes linked to MTZ activation machinery.

Collectively, these data indicate that MTZ–MMC synergy is accompanied by coordinated metabolic reprogramming involving enhanced NADPH-generating pathways and modulation of MTZ-associated redox enzymes. This metabolic shift is consistent with compensatory responses to thioredoxin system disruption and combined redox and genotoxic stress.

## 4 Discussion

MTZ remains the primary therapy for trichomoniasis; however, the emergence of resistant *T. vaginalis* isolates increasingly compromises treatment efficacy. MTZ is a prodrug activated within the parasite hydrogenosome, where enzymes such as PFOR and ferredoxin transfer electrons to the nitro group of MTZ, generating reactive intermediates that damage DNA and other cellular components (Leitsch, 2019; Tachezy et al., 2022). Alterations in these redox-dependent activation pathways have therefore been widely implicated in MTZ resistance (Leitsch, 2019).

Beyond hydrogenosomal activation, parasite redox buffering systems may also influence responses to MTZ-induced stress. *T. vaginalis* relies primarily on the Trx–Prx redox network to maintain intracellular redox homeostasis (Ellis et al., 1994; Müller et al., 2003). Nitroimidazole drugs can be reduced by TrxR, linking Trx-mediated redox processes to nitroimidazole toxicity and cellular redox disruption (Leitsch et al., 2009). Consistent with this role, pharmacological inhibition of TrxR, such as by the gold-containing compound auranofin, disrupts parasite redox homeostasis, highlighting the thioredoxin system as a potential therapeutic vulnerability in *T. vaginalis*. In line with this concept, our results show that MMC significantly suppresses TrxR activity in MTZ-resistant isolates. While auranofin remains a benchmark inhibitor for redox disruption, our findings suggest that MMC may represent another clinically relevant compound capable of interfering with TrxR-mediated redox buffering and enhancing susceptibility of resistant parasites to nitroimidazole treatment (Tejman-Yarden et al., 2013).

In the present study, we investigated whether MTZ-associated cytotoxicity in *T. vaginalis* involves ferroptosis-related mechanisms. Because intracellular iron availability has been reported to influence MTZ susceptibility (Elwakil et al., 2017), we hypothesized that MTZ-induced parasite death might involve ferroptosis-like pathways. However, our results did not support this model. Although ferroptosis inducers reduced parasite viability, inhibition of ferroptosis or iron chelation failed to restore parasite survival, and lipid peroxidation markers were not significantly increased, indicating that MTZ cytotoxicity does not follow canonical ferroptotic mechanisms.

Instead, transcriptional analyses revealed activation of Trx-associated antioxidant responses following exposure to MTZ and ferroptosis-inducing agents. Both MTZ-S and MTZ-R parasites showed induction of Trx-related transcripts, consistent with drug-induced oxidative stress. Notably, the MTZ-R isolate exhibited stronger induction of TrxR relative to Trx, suggesting that resistant parasites may rely more heavily on TrxR-mediated redox buffering during MTZ challenge.

To examine the functional relevance of TrxR, we inhibited its activity using MMC, a compound known to induce DNA damage and interfere with Trx-dependent redox systems (Paz et al., 2012). In our study, MMC significantly suppressed TrxR enzymatic activity and enhanced the antiparasitic effect of MTZ in the resistant isolate, producing a clear synergistic interaction. These findings indicate that disruption of TrxR-mediated redox buffering can sensitize MTZ-resistant parasites to MTZ treatment. We next evaluated AF, a well-characterized TrxR inhibitor, to determine whether selective TrxR inhibition alone could account for this phenotype. However, neither AF monotherapy nor AF–MTZ co-treatment significantly inhibited MTZ-R parasite growth (Fig. S3), suggesting that selective TrxR inhibition alone is insufficient to fully reproduce the enhanced activity observed during MMC–MTZ co-treatment. Interestingly, synergistic interactions between MMC and MTZ have also been reported in *Clostridioides difficile*, where MMC was proposed to potentiate MTZ activity by increasing DNA damage and amplifying antimicrobial stress responses (Gong et al., 2025). Although the molecular targets differ from those identified in *T. vaginalis*, findings from both studies support the concept that MMC–MTZ co-treatment enhances nitroimidazole efficacy through complementary mechanisms. Collectively, these observations suggest that while TrxR-associated redox regulation contributes to MTZ susceptibility, the synergistic interaction between MMC and MTZ is unlikely to be explained solely by TrxR inhibition and may instead arise from the combined disruption of multiple cellular stress-response pathways.

Consistent with this redox perturbation, MTZ–MMC co-treatment was also associated with transcriptional changes in metabolic pathways linked to cellular redox homeostasis. Genes involved in glycolysis and the PPP were differentially regulated following combination treatment. Because the PPP provides NADPH required to sustain Trx-dependent antioxidant systems (Lu and Holmgren, 2014), these changes likely represent compensatory metabolic responses to MMC-induced redox and genotoxic stress.

Several limitations should also be considered. First, the present analysis was performed using a limited number of parasite isolates, and additional clinical strains will be required to determine whether TrxR-associated responses are broadly conserved among MTZ-resistant parasites. Second, although MMC suppressed TrxR activity and enhanced MTZ susceptibility, MMC is known to have multiple cellular targets. Future studies employing genetic approaches or more specific TrxR inhibitors will be necessary to further validate the role of the Trx system in MTZ resistance.

Together, these findings support a model in which MTZ exposure triggers oxidative stress responses in *T. vaginalis*, leading to activation of Trx-associated redox pathways. The pronounced induction of TrxR observed in MTZ-R parasites, together with the synergistic interaction between MTZ and MMC, suggests that TrxR-mediated redox buffering contributes to parasite tolerance under MTZ challenge. Targeting Trx-dependent redox homeostasis may therefore represent a potential strategy to enhance MTZ efficacy against resistant *T. vaginalis*.

## 5 Conclusion

In this study, we investigated whether MTZ-induced cytotoxicity in *T. vaginalis* involves ferroptosis-related mechanisms and explored alternative pathways associated with MTZ resistance. Our results demonstrate that although ferroptosis-inducing agents reduce parasite viability, MTZ-associated killing does not exhibit canonical ferroptotic features. Instead, MTZ-resistant parasites display selective induction of TrxR under drug stress, suggesting an adaptive redox-buffering mechanism. Pharmacological inhibition of TrxR by MMC significantly enhanced MTZ efficacy and produced a synergistic antiparasitic effect in resistant isolates. Although selective TrxR inhibition alone was insufficient to fully reproduce this phenotype, our findings support a role for thioredoxin-dependent redox regulation in MTZ susceptibility. Collectively, these results identify the thioredoxin system as a resistance-associated vulnerability and suggest that targeting redox homeostasis may represent a promising strategy for overcoming MTZ resistance in *T. vaginalis*.

## Availability of data and materials

All data generated or analyzed during this study are included in this published article and its supplementary information files.

## Funding

This work was supported by the 10.13039/100020595National Science and Technology Council, Taiwan (10.13039/100020595NSTC 114-2221-E−182-044 to P.J.H.; 10.13039/100020595NSTC 113-2320-B-006-033 to W.H.C.) and by 10.13039/501100005795Chang Gung Memorial Hospital, Linkou (CMRPG3N0201 to Y.M.Y.).

## CRediT authorship contribution statement

**Yuan-Ming Yeh:** Conceptualization, Writing – review & editing. **Po-Jung Huang:** Conceptualization, Writing – review & editing. **Yu-Lo Wu:** Data curation, Formal analysis. **Chun-Hsien Chen:** Conceptualization, Writing – review & editing. **Pei-Yun Chen:** Data curation, Formal analysis. **Yu-Tzu Hsu:** Data curation, Formal analysis. **Wei-Hung Cheng:** Conceptualization, Writing – original draft.

## Declaration of competing interest

The authors declare the following financial interests/personal relationships which may be considered as potential competing interests: Wei-Hung Cheng reports financial support was provided by National Science and Technology Council, Taiwan. Po-Jung Huang reports financial support was provided by National Science and Technology Council, Taiwan. Yuan-Ming Yeh reports financial support was provided by Linkou Chang Gung Memorial Hospital. If there are other authors, they declare that they have no known competing financial interests or personal relationships that could have appeared to influence the work reported in this paper.
