# Supplementary material for: Mitomycin C potentiates metronidazole activity in resistant Trichomonas vaginalis through suppression of thioredoxin reductase
Source: Int J Parasitol Drugs Drug Resist. 2026 Jul 18;31:100661. doi: 10.1016/j.ijpddr.2026.100661 (PMC13393407; doi:10.1016/j.ijpddr.2026.100661)
Supplement: Multimedia component 1 [file mmc1.pdf]

Table S1. Primers used in this study

Accession Number	Primer Name	Direction (F/R)	Sequence (5'→3')
TVAGG3_0062590	Ribosomal protein L8	F	TTGCGGTATCAAGATGAACCCAG
		R	GAACCAAAGCTTTATGCAAGGTTGT
TVAGG3_0707900	TvGPX	F	ACCTCGAAGCCCTTTACCAA
		R	TGCCTGGCTCTTGGGATAAA
TVAGG3_0344710	TvTrxR	F	ACAAGACAGGCGAGACACAG
		R	CCTGGCGGTATGTTCTGTCA
TVAGG3_0939120	TvTrx	F	CGGTCCATGCCAACGCCTTG
		R	GGCAACATCAGCACCAACAAAC
TVAGG3_0657800	TvTrx2	F	GACTTCTTTGCTACATGGTGCGGC
		R	TGGAACAGACTGAACGCCGAAA
TVAGG3_0322530	TvPFK	F	TGCAGTTCTCTCTAGTGGCC
		R	CACGGAAGCCACCAGTAATG
TVAGG3_0120590	TvALDO	F	AAGTCACTCGGTCTCTGCAA
		R	TTGACGGAGGCTGTGATGAT
TVAGG3_0815950	TvTPI	F	GGCAAGTGGGACGATGTTG
		R	TTAGCAGCAAGGATGTCACG
TVAGG3_0303510	TvGAPDH	F	CCAAGTTGTCGCTATCCACG
		R	TGCTTAGCCTCATCGACTGT
TVAGG3_1048680	TvENOL	F	ACAGGTGTTGGTGAAGCTCT
		R	AGCACATTCCCTTGAGAGCT
TVAGG3_0205560	TvG6PD	F	ATTCTCACGTCTCCACCAGG
		R	GTCATCGTAGCCACCAGAGA
TVAGG3_0982660	Tv6PGDH	F	CGATGGTGGCAACTCTCACT
		R	CTCTTCACCGCCGGAGATAC
TVAGG3_0581060	TvCK	F	TACAACAGGAGCCGGAGATG
		R	AGCAGCACAACCTCTCTTTG
TVAGG3_0961100	TvTK	F	GGAGTAAGACTTGGCTGGGA
		R	CTCTTCACCGCCGGAGATAC
TVAGG3_0155570	TvTALDO	F	TCCTCAAGATTGTCCCAGGC
		R	CGTTCTGCACATTTCTCTGGT
TVAGG3_0998520	TvPFOR	F	CCAGATCACACCACTCGACT
		R	TCTTGATTCCGGCTTCGTGA
TVAGG3_0548570	TvNADHOX	F	ATTGGCTTGGCGTCCTTGAT
		R	TTCCCAGTTCTTGCCCTCTT
TVAGG3_0751360	TvPFOR2	F	GAGGCCATCTGCAAGAACCT
		R	TCGACGAGAACTGCACCTTC
TVAGG3_0170920	TvFeS	F	GCTCCATCTACAGGCAAGCA
		R	ATGGCTGTCTCTGGGTTGTG
TVAGG3_0517720	TvFeS2	F	AATTGAACCACGCTGCTTGC
		R	GTAACTTTGTGGCCCCGAGA

TVAGG3_0565200	TvFlavo	F	AGAACGCCAAGGTCCTCATC
		R	CCGAAGTGGCCGAATGTTTG
TVAGG3_0389530	TvFlavo2	F	TCCCAATCTGGTGGATGGGT
		R	TGCCATCACCGTTCCAAAGT
