# Supplementary material for: Mitomycin C potentiates metronidazole activity in resistant Trichomonas vaginalis through suppression of thioredoxin reductase
Source: Int J Parasitol Drugs Drug Resist. 2026 Jul 18;31:100661. doi: 10.1016/j.ijpddr.2026.100661 (PMC13393407; doi:10.1016/j.ijpddr.2026.100661)
Supplement: Multimedia component 2 [file mmc2.pdf]

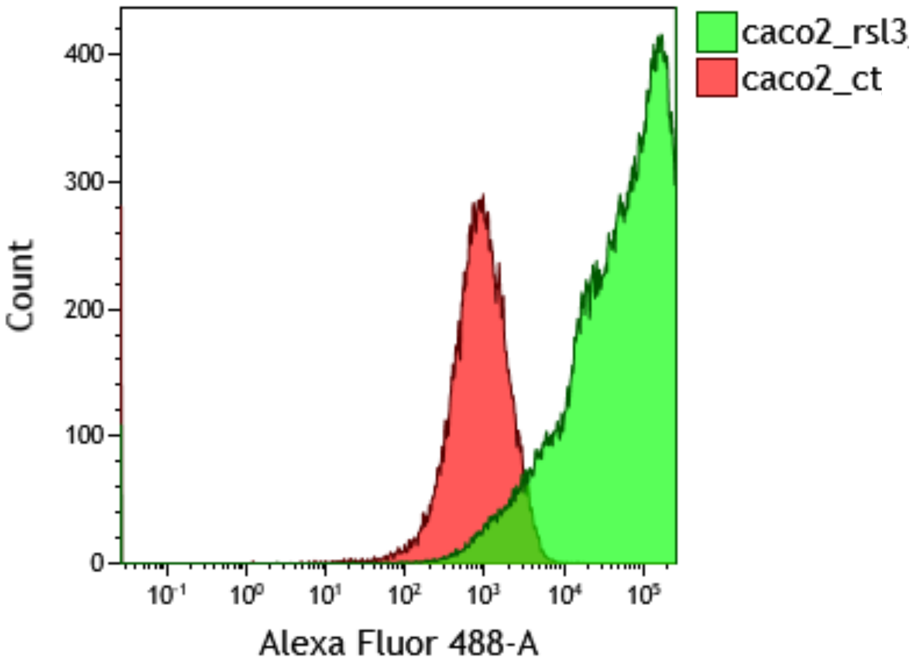

Supplementary Figure S1. RSL3-induced lipid peroxidation in Caco-2 cells.

Caco-2 cells were treated with RSL3 or vehicle control before fluorescent detection. Following treatment, cells were incubated with 2 μM C11-BODIPY 581/591 for 30 min at 37°C in the dark. After washing with PBS, cells were analyzed by flow cytometry. Oxidation of C11-BODIPY was measured as increased fluorescence intensity in the Alexa Fluor 488-A (FITC) channel. Representative overlaid histograms are shown, with control cells displayed in red and RSL3-treated cells in green. Fluorescence intensity is presented on a logarithmic scale. Data are representative of three independent experiments.
