# Supplementary material for: Mitomycin C potentiates metronidazole activity in resistant Trichomonas vaginalis through suppression of thioredoxin reductase
Source: Int J Parasitol Drugs Drug Resist. 2026 Jul 18;31:100661. doi: 10.1016/j.ijpddr.2026.100661 (PMC13393407; doi:10.1016/j.ijpddr.2026.100661)
Supplement: Multimedia component 3 [file mmc3.pdf]

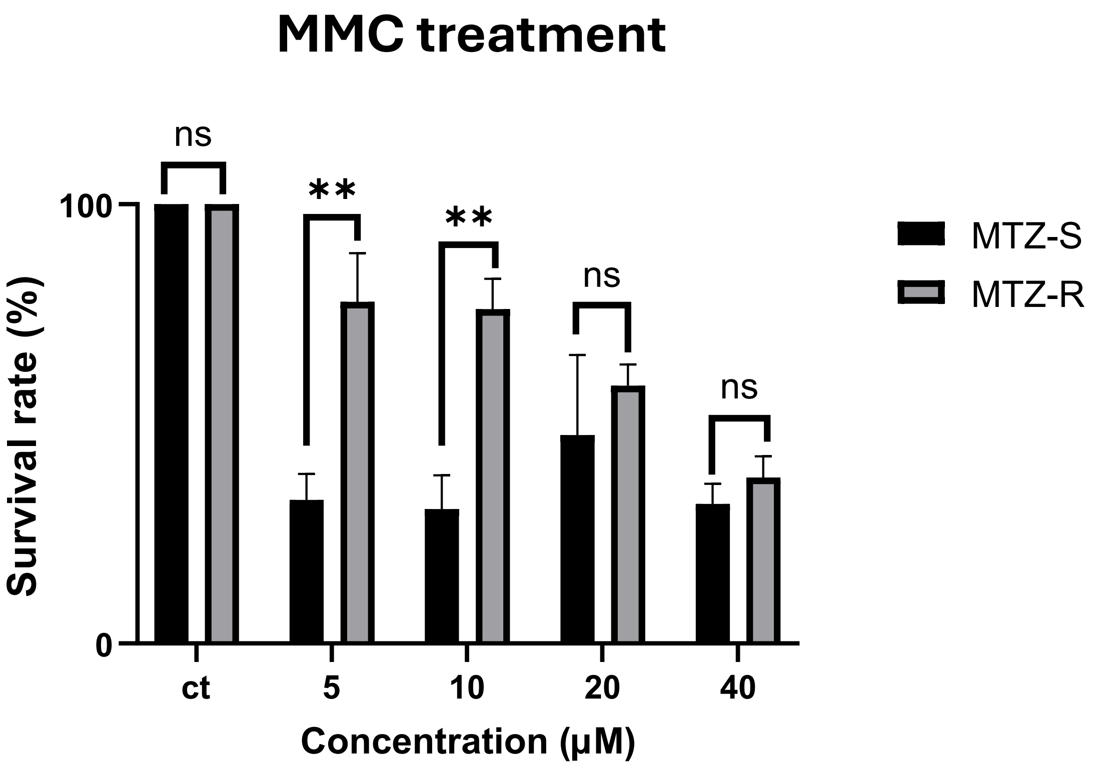

Supplementary Figure S2. MMC sensitivity in MTZ-sensitive and MTZ-resistant *T. vaginalis*.

Sensitivity of MTZ-sensitive (MTZ-S, black bars) and MTZ-resistant (MTZ-R, gray bars) *T. vaginalis* isolates to MMC treatment. Parasites were treated with the indicated concentrations of MMC for 18 h, and survival rates were determined relative to vehicle-treated controls (ct). Data represent mean ± SD from three independent experiments. Statistical analysis was performed using unpaired two-tailed Student’s t-test. ns, not significant; **$p < 0.01$.
