# Supplementary material for: Mitomycin C potentiates metronidazole activity in resistant Trichomonas vaginalis through suppression of thioredoxin reductase
Source: Int J Parasitol Drugs Drug Resist. 2026 Jul 18;31:100661. doi: 10.1016/j.ijpddr.2026.100661 (PMC13393407; doi:10.1016/j.ijpddr.2026.100661)
Supplement: Multimedia component 4 [file mmc4.pdf]

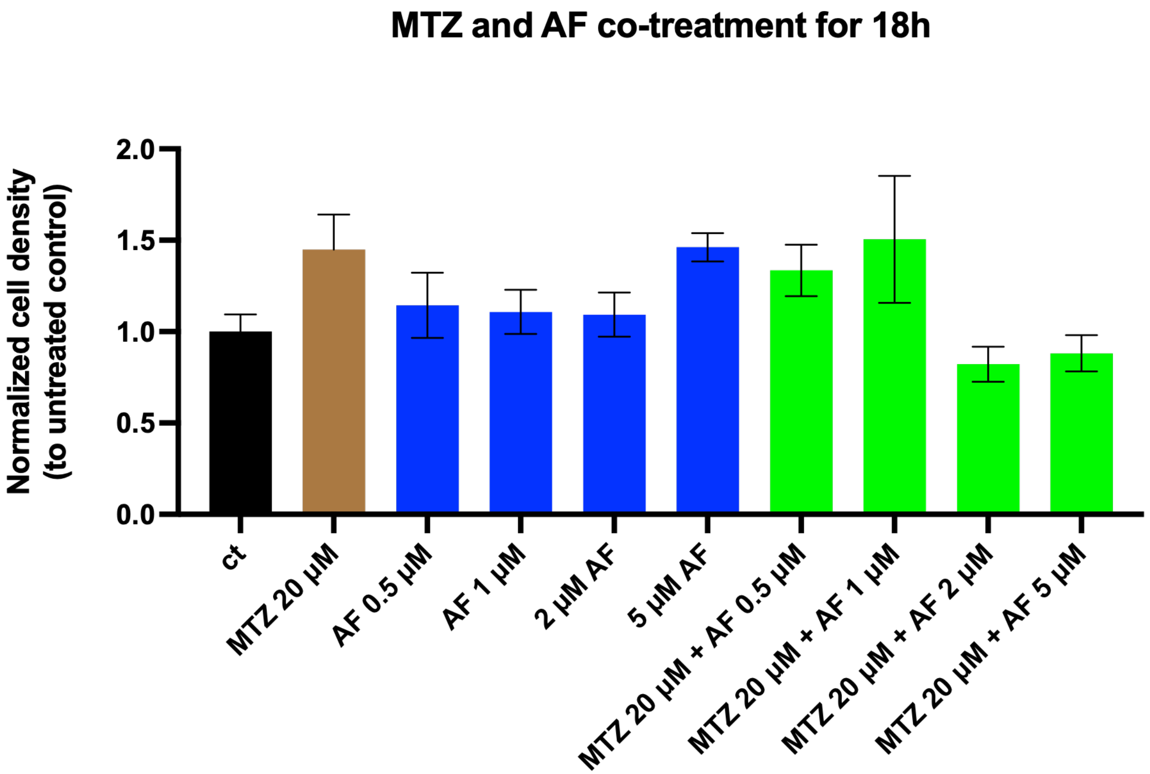


**Supplementary Figure S3. Effects of auranofin alone and in combination with metronidazole on MTZ-resistant *T. vaginalis*.**

MTZ-resistant (MTZ-R) parasites were treated with auranofin (AF; 0.5, 1, 2, or 5 µM), metronidazole (MTZ; 20 µM), or their combinations for 18 h. The group labeled "0" represents the untreated vehicle control. Parasite growth was determined by cell density measurement after treatment. AF treatment alone did not significantly affect parasite growth under the tested conditions. Co-treatment with MTZ and AF at 2 or 5 µM showed a tendency toward reduced parasite growth compared with MTZ alone; however, the differences were not statistically significant. Data are presented as mean ± SD from three independent experiments.
